# Use of Bimatoprost Intracameral Implant for the Treatment of Glaucoma: A Focused Narrative Review

**DOI:** 10.7759/cureus.104676

**Published:** 2026-03-04

**Authors:** Arham Yahya Rizwan Khan

**Affiliations:** 1 Medicine and Surgery, Shifa Tameer-e-Millat University, Islamabad, PAK

**Keywords:** bimatoprost, bimatoprost intracameral implant, glaucoma, intraocular pressure (iop), ocular hypertension (oht), open-angle glaucoma

## Abstract

The objective of this focused narrative review is to summarize clinical and preclinical evidence on intracameral bimatoprost implants for adults with open-angle glaucoma (OAG) or ocular hypertension (OHT), focusing on comparative efficacy, safety, and adherence-related outcomes. PubMed/MEDLINE and Google Scholar were searched from inception through January 31, 2026, using combinations of the terms bimatoprost implant, bimatoprost sustained-release, bimatoprost SR, Durysta, glaucoma, and ocular hypertension. Eligible reports included human clinical trials or observational studies evaluating any intracameral bimatoprost implant dose in OAG or OHT, and relevant nonclinical pharmacokinetic or pharmacodynamic studies. Conference abstracts without full text and non-English reports were excluded. Records were screened at the title/abstract level followed by full-text review, and data were extracted on intraocular pressure change, need for rescue therapy or additional procedures, treatment-emergent adverse events (TEAEs), and corneal endothelial cell density (CECD).

Across phase I/II and phase III trials, bimatoprost implants produced intraocular pressure reductions comparable to topical timolol or topical bimatoprost through early follow-up, and many eyes remained drop-free for months to a year. Corneal adverse events and CECD loss were infrequent after single administration but increased with fixed-interval repeat dosing, with a less favorable profile at higher dose strengths. It was concluded that intracameral bimatoprost implants offer a drop-sparing alternative that may reduce adherence burden, but careful patient selection and monitoring for corneal endothelial effects are central to safe use.

## Introduction and background

Glaucoma is a chronic optic neuropathy in which lowering intraocular pressure (IOP) reduces the risk of progression and vision loss in open-angle glaucoma (OAG) and ocular hypertension (OHT) [1-6]. Most patients, therefore, require long-term IOP-lowering therapy, often for years.

Topical eye drops remain the most common first-line approach, but real-world adherence and correct drop administration are inconsistent. Reported nonadherence is common and is linked to IOP fluctuation and worse visual field outcomes [7-12]. Barriers include forgetfulness, side effects, complex regimens, cost, and limitations related to age or disability [10-16]. These challenges have driven interest in sustained drug delivery approaches that reduce the daily drop burden.

Prostaglandin analogs are widely used because of their favorable efficacy and systemic safety, but chronic topical exposure can contribute to ocular surface symptoms and periocular effects [13-16]. Intracameral prostaglandin delivery is novel because it aims to provide durable IOP lowering while reducing reliance on daily drops. The biodegradable intracameral bimatoprost implant delivers the drug directly to the anterior segment target tissues and may reduce off-target exposure based on nonclinical distribution studies [17-21]. The key clinical question is the balance between efficacy and durability versus corneal endothelial safety, particularly changes in corneal endothelial cell density (CECD) with repeat dosing [22,23]. Accordingly, this focused narrative review summarizes clinical and supportive nonclinical evidence on intracameral bimatoprost implants in adults with open angle glaucoma or ocular hypertension, emphasizing comparative efficacy, durability, and corneal endothelial safety.

Methods

Review Type and Reporting Approach

This article is a focused narrative review. A structured literature search was used to identify key clinical and supportive nonclinical evidence for narrative synthesis. Preferred Reporting Items for Systematic Reviews and Meta-Analyses (PRISMA) reporting was not followed, a study selection flow diagram was not prepared, and no validated study level risk of bias tool was applied. For this reason, findings are interpreted qualitatively, with emphasis on clinical relevance and key limitations.

Literature Search Strategy

PubMed/MEDLINE and Google Scholar were searched from database inception through January 31, 2026. The PubMed search string was: (“bimatoprost implant” OR “bimatoprost sustained release” OR “bimatoprost sustained-release” OR “bimatoprost SR” OR Durysta) AND (glaucoma OR “ocular hypertension”) AND (intracameral OR implant OR sustained-release). The search was supplemented by manual screening of reference lists of included articles and relevant background reviews.

Scope and Inclusion Approach

We included randomized trials, prospective studies, and observational cohort studies or case series with at least 10 eyes evaluating intracameral bimatoprost implants of any dose in adults with OAG or OHT. We also included nonclinical pharmacokinetic or pharmacodynamic studies that informed tissue distribution or mechanistic plausibility. We excluded studies that did not evaluate intracameral implants, pediatric populations, non-English full texts, and conference abstracts without sufficient methods and outcomes for appraisal. Nonclinical animal studies were included only to support mechanistic plausibility and ocular tissue distribution, and clinical conclusions in this review are based on human studies.

Study Screening and Narrative Synthesis Approach

Titles and abstracts were screened to identify studies most relevant to intracameral bimatoprost implants in adults with OAG or OHT. Full texts were reviewed when eligibility or outcome reporting required clarification. Data were extracted on study design, implant dose and retreatment approach, comparator when applicable, IOP outcomes, need for rescue therapy or additional procedures, and treatment emergent adverse events with emphasis on corneal findings and CECD.

Study Limitations and Confidence in Evidence

Given the heterogeneity in dosing regimens, follow-up duration, and outcome definitions, a meta analysis was not performed. No validated risk of bias tool was applied and no study level quality ratings were generated, consistent with the focused narrative design. Instead, key limitations were considered qualitatively, including masking and follow-up completeness in randomized trials, and confounding, selection, and outcome ascertainment in observational studies. The ARTEMIS trials [22,23] were masked and had high completion rates but were sponsor funded, while real-world studies were more susceptible to selection bias and confounding.

## Review

Evidence base and key clinical trade-off

The intracameral bimatoprost implant 10 µg was authorized in March 2020 for IOP lowering in adults with OAG or OHT [19]. The clinical evidence base includes phase I/II studies and phase III trials comparing implant dosing strategies with topical timolol, as well as a paired-eye trial comparing the implant with selective laser trabeculoplasty [18,22-25]. Real-world cohort studies also report durability and safety outcomes after single administration in routine practice settings [26,27]. Across these sources, the main clinical trade-off is durability and drop reduction versus corneal endothelial safety, which appears dependent on the dose or retreatment [22,23,25].

Comparative efficacy and durability

Across trials, early IOP lowering with the implant was broadly comparable to topical comparators, while durability varied across follow-up intervals and retreatment approaches [18,22-25]. Many eyes remained drop free for months, but waning effect over time was reported in a subset, which is clinically relevant when planning monitoring and rescue strategies [18]. Comparative efficacy and drop burden outcomes are summarized in Table 1.

**Table 1:** Comparative efficacy and medication-burden outcomes across intracameral bimatoprost implant studies OAG: open-angle glaucoma; OHT: ocular hypertension; IOP: intraocular pressure; SLT: selective laser trabeculoplasty; PGA: prostaglandin analog.

Study (ref)	Design and population	Implant dose and regimen	Comparator	Key efficacy outcome(s)	Drop-burden or rescue outcome(s)
ARTEMIS 1 [22]	Phase III, 20-month, multicenter trial in OAG/OHT	10 µg or 15 µg on day 1, week 16, week 32	Timolol 0.5% twice daily	Noninferior IOP lowering through week 12; mean diurnal IOP reduced to 16.5–17.2 (10 µg) and 16.5–17.0 (15 µg) mmHg through week 12	Many eyes remained controlled without additional therapy for prolonged periods after implant administration
Pooled phase III single-administration analysis [18]	Pooled ARTEMIS datasets evaluating single 10 µg administration through week 15	10 µg on day 1 only	Timolol 0.5% twice daily	≥20% IOP reduction in 71.9% at week 12 and 57.0% at week 15 (hour 0)	2.1% required rescue before week 15
ARTEMIS 2 [23]	Phase III, 20-month trial in OAG/OHT	10 µg or 15 µg on day 1, week 16, week 32	Timolol 0.5% twice daily	Mean IOP reductions through week 12 ranged 6.2–7.4 (10 µg), 6.5–7.8 (15 µg), and 6.1–6.7 mmHg (timolol)	Reached month 20 without rescue: 64.8% (10 µg), 64.2% (15 µg), 78.4% (timolol)
Phase I/II 24-month study [24]	Phase I/II study with extended follow-up	Single administration across dose groups, including 10 µg	Fellow-eye topical bimatoprost 0.03% once daily	≥20% IOP reduction at week 12 was 76.2% for 10 µg; durability varied across follow-up	A subset remained without additional IOP-lowering treatment for up to 24 months after a single implant
Implant 10 µg vs SLT paired-eye trial [25]	Phase III, 24-month paired-eye trial	Up to 2 implant administrations with safety-based retreatment scheduling	SLT 360° in contralateral eye	IOP reduction at weeks 4, 12, 24: 6.8, 6.9, 6.9 mmHg (implant) vs 6.2, 6.4, 6.5 mmHg (SLT)	Probability of no rescue at days 360 and 720: 67.5% and 50.2% (implant) vs 68.7% and 60.6% (SLT)
Real-world cohort (105 patients) [26]	Retrospective real-world study in OAG/OHT	Typically single 10 µg implant	None	Clinically maintained IOP control in routine care context	23.8% completed 24 months without second implant or rescue therapy
PGA replacement cohort (129 eyes) [27]	Retrospective cohort replacing topical PGAs with implant	Single implant replacing topical PGA	Prior topical PGA therapy	Maintained IOP control after switch in many eyes	Median time without topical therapy reported; 40.5% remained intervention-free at 12 months and 27.8% remained medication-free

Treatment-emergent adverse events (TEAEs) such as excessive eyelash development, eyelash pigmentation, skin pigmentation, or periorbital fat atrophy are often observed with glaucoma medications. In the ARTEMIS 1 trial, iritis and corneal endothelial cell loss were higher in the bimatoprost group, particularly in the 15 μg concentration. The authors concluded that the lower bimatoprost concentration indicated a favorable risk-benefit assessment [22]. These findings align with the findings of a phase I/II study of bimatoprost implants, conducted over two years, which found that TEAEs such as iris pigmentation, eyelash growth, and periorbital fat atrophy were observed in eyes treated with topical bimatoprost but not observed in eyes treated with bimatoprost implants [24]. These clinical findings have been strengthened by nonclinical drug distribution investigations conducted on dogs, wherein drug levels in the periocular and ocular surfaces following the injection of a bimatoprost implant were either negligible or reduced in comparison to drug levels following topical administration of 0.03% bimatoprost [21,22].

Targeted drug delivery via intracameral implantation reduces periocular exposure and may lessen eyelash and skin pigmentation and ocular surface adverse effects that can limit chronic topical prostaglandin analog use [17]. In routine practice, the implant is indicated as a single administration per eye, largely because corneal events increased with fixed-interval repeat dosing in phase III programs [19]. In ARTEMIS 1, the incidence of a ≥20% decrease in CECD after three administrations at fixed 16-week intervals was 10.2% with the 10 μg implant and 21.8% with the 15 μg implant, supporting a dose-dependent corneal safety signal and a more favorable benefit-risk profile for the 10 μg dose [22].

In ARTEMIS 2, both 10 μg and 15 μg implants met the primary non-inferiority endpoint versus timolol, but ocular TEAEs were more frequent with the implant and were dose dependent [23]. Specular microscopy demonstrated a time-dependent reduction in CECD, with mean CECD at month 20 approximately 5% below baseline in the 10 μg group compared with approximately 1% below baseline in the timolol group. The proportion of study eyes with a ≥20% CECD decrease at month 20 was 8.1% with the 10 μg implant and 24.4% with the 15 μg implant, compared with 0.6% with timolol [23]. Collectively, these findings support a more favorable benefit-risk profile for the 10 μg dose and motivate evaluation of alternative retreatment schedules intended to reduce corneal risk [23]. Key safety outcomes, including corneal findings, are summarized in Table 2.

**Table 2:** Safety outcomes and corneal endothelial findings across key clinical studies IOP: intraocular pressure; SLT: selective laser trabeculoplasty; PGA: prostaglandin analog; TEAE: treatment-emergent adverse events; CECD: corneal endothelial cell density.

Study (ref)	Dose and regimen	Key TEAEs reported in the context of the manuscript	Corneal findings and CECD	Practical takeaway
ARTEMIS 1 [22]	10 µg or 15 µg, readministration at fixed 16-week intervals	Higher corneal and inflammatory TEAEs vs timolol; more frequent with 15 µg	≥20% CECD loss after 3 administrations: 10.2% (10 µg) vs 21.8% (15 µg)	Corneal risk increases with repeat dosing and higher dose
ARTEMIS 2 [23]	10 µg or 15 µg, fixed readministration	Treatment-related TEAEs more frequent with implant and dose dependent	Mean CECD at month 20 approximately 5% below baseline (10 µg) vs approximately 1% (timolol); ≥20% CECD decrease at month 20: 8.1% (10 µg) vs 24.4% (15 µg) vs 0.6% (timolol)	Supports preference for 10 µg and careful corneal monitoring
Single-administration analysis [18]	Single 10 µg administration	Favorable short-term safety profile reported in pooled analyses	Mean CECD change after single administration in phase I/II dataset: −111.5 cells/mm², mean −4.2%	Corneal signal is smaller after single administration than after fixed repeat dosing
Implant 10 µg vs SLT paired-eye trial [25]	Up to 2 implant administrations with safety-based scheduling	Increased IOP attributed to wearing off of efficacy described as common ocular TEAE	Mean % CECD change at month 24: −6.2% (implant) vs −3.1% (SLT); fixed retreatment worse than flexible scheduling	Flexible retreatment appears to improve safety profile
Real-world cohort (105 patients) [26]	Typical single 10 µg use in practice	No implant removal; no corneal edema reported in this cohort	No serious corneal safety signal reported	Real-world safety appears acceptable with careful use
PGA replacement cohort (129 eyes) [27]	Single implant replacing topical PGA	Anterior chamber inflammation and corneal edema described as uncommon and transient	Corneal events were not a dominant limitation in this cohort	Supports feasibility of drop-sparing switch in selected patients

Clinical Interpretation of the CECD Changes

The corneal endothelium has limited regenerative capacity, so even modest mean CECD reductions can be clinically relevant in eyes with low baseline endothelial reserve. Baseline and follow-up specular microscopy can be considered, particularly in patients with prior intraocular surgery, corneal dystrophy, a history of uveitis, or a narrow iridocorneal angle. Avoiding repeat administration at fixed short intervals and ensuring implant positioning away from the corneal endothelium appear central to mitigating risk.

Real-world cohort studies suggest that a subset of patients remain free of additional topical therapy or procedures for months after a single 10 µg implant, with low rates of serious corneal events reported in routine practice [26,27]. These findings support the drop-sparing potential of the implant in selected patients, but this interpretation is limited by retrospective design, heterogeneous prior treatments, and nonstandardized follow-up. Key durability and safety outcomes from real-world cohorts are summarized in Tables 1, 2.

Nonclinical Evidence

Nonclinical studies support mechanistic plausibility and tissue distribution consistent with intracameral delivery. In animal models, intracameral sustained release approaches have been associated with higher local bimatoprost exposure and longer IOP lowering than topical administration in controlled settings [28,29]. These findings provide supportive context, but clinical conclusions regarding efficacy, durability, and corneal endothelial safety should be based on human trials and real-world studies.

Future recommendations

To ensure the safety of the bimatoprost implant in clinical practice, prospective longitudinal studies evaluating the incidence of adverse events, such as corneal endothelial cell loss, raised IOP, and intraocular inflammation, are crucial for characterizing the implant's safety profile. The effect of the bimatoprost intracameral implant on patients' quality of life, visual function, and satisfaction with treatment should be thoroughly assessed. Clinical trials and observational research with patient-reported outcome measures will yield important information on how the implant affects the general health and treatment experience of patients. To optimize the effectiveness and safety of the bimatoprost intracameral implant, optimization of the treatment protocols, including dose schedules, implant insertion methods, and supplementary treatments, is necessary. Extensive observational research and registry-based evaluations will provide a comprehensive understanding of the implant's practical effectiveness and therapeutic trends across diverse patient demographics and healthcare settings. To guide healthcare decision-making and resource allocation, studies comparing the cost-effectiveness of the bimatoprost intracameral implant with alternative glaucoma treatments are crucial. Achieving these research goals in the future will improve patient outcomes by enhancing our understanding of the role of bimatoprost intracameral implant in treating glaucoma and maximizing its therapeutic potential.

## Conclusions

The use of bimatoprost intracameral implants is a potential new treatment option for individuals with glaucoma. A thorough analysis of the literature reveals that bimatoprost implants are highly effective in reducing IOP. Furthermore, the implant's sustained-release mechanism ensures continuous medication delivery, resulting in sustained IOP-lowering benefits over a longer period. Most adverse effects associated with bimatoprost implants are mild and temporary and usually subside with the right care and monitoring.

The sustained-release formulation of the implant removes the requirement for daily topical administration, streamlines the treatment plans, and perhaps enhances patients’ adherence to the treatment. However, to guarantee the implant's continued safety in clinical practice and to further understand its safety profile, prolonged monitoring and surveillance are crucial. The implant has significant potential to improve outcomes and raise the standard of care for those with this disabling eye illness, but further study is necessary to optimize its clinical usage and enhance its function in managing glaucoma.

## References

[1] Flaxman SR, Bourne RRA, Resnikoff S (2017). Global causes of blindness and distance vision impairment 1990-2020: a systematic review and meta-analysis. Lancet Glob Health.

[2] Allison K, Patel D, Alabi O (2020). Epidemiology of glaucoma: the past, present, and predictions for the future. Cureus.

[3] Jonas JB, Aung T, Bourne RR, Bron AM, Ritch R, Panda-Jonas S (2017). Glaucoma. Lancet.

[4] Baneke AJ, Aubry J, Viswanathan AC, Plant GT (2020). The role of intracranial pressure in glaucoma and therapeutic implications. Eye (Lond).

[5] Gordon MO, Kass MA (2018). What we have learned from the ocular hypertension treatment study. Am J Ophthalmol.

[6] Kass MA, Heuer DK, Higginbotham EJ (2002). The ocular hypertension treatment study: a randomized trial determines that topical ocular hypotensive medication delays or prevents the onset of primary open-angle glaucoma. Arch Ophthalmol.

[7] Robin AL, Muir KW (2019). Medication adherence in patients with ocular hypertension or glaucoma. Expert Rev Ophthalmol.

[8] Newman-Casey PA, Blachley T, Lee PP, Heisler M, Farris KB, Stein JD (2015). Patterns of glaucoma medication adherence over four years of follow-up. Ophthalmology.

[9] Newman-Casey PA, Niziol LM, Gillespie BW, Janz NK, Lichter PR, Musch DC (2020). The association between medication adherence and visual field progression in the collaborative initial glaucoma treatment study. Ophthalmology.

[10] McClelland JF, Bodle L, Little JA (2019). Investigation of medication adherence and reasons for poor adherence in patients on long-term glaucoma treatment regimes. Patient Prefer Adherence.

[11] Newman-Casey PA, Robin AL, Blachley T, Farris K, Heisler M, Resnicow K, Lee PP (2015). The most common barriers to glaucoma medication adherence: a cross-sectional survey. Ophthalmology.

[12] Tapply I, Broadway DC (2021). Improving adherence to topical medication in patients with glaucoma. Patient Prefer Adherence.

[13] Tang W, Zhang F, Liu K, Duan X (2019). Efficacy and safety of prostaglandin analogues in primary open-angle glaucoma or ocular hypertension patients: a meta-analysis. Medicine (Baltimore).

[14] Li T, Lindsley K, Rouse B (2016). Comparative effectiveness of first-line medications for primary open-angle glaucoma: a systematic review and network meta-analysis. Ophthalmology.

[15] Dreer LE, Girkin CA, Campbell L, Wood A, Gao L, Owsley C (2013). Glaucoma medication adherence among African Americans: program development. Optom Vis Sci.

[16] Schwartz GF, Hollander DA, Williams JM (2013). Evaluation of eye drop administration technique in patients with glaucoma or ocular hypertension. Curr Med Res Opin.

[17] Lewis RA, Christie WC, Day DG (2017). Bimatoprost sustained-release implants for glaucoma therapy: 6-month results from a phase I/II clinical trial. Am J Ophthalmol.

[18] Medeiros FA, Sheybani A, Shah MM (2022). Single administration of intracameral bimatoprost implant 10 µg in patients with open-angle glaucoma or ocular hypertension. Ophthalmol Ther.

[19] Sirinek PE, Lin MM (2022). Intracameral sustained release bimatoprost implants (Durysta). Semin Ophthalmol.

[20] Shen J, Robinson MR, Struble C, Attar M (2020). Nonclinical pharmacokinetic and pharmacodynamic assessment of bimatoprost following a single intracameral injection of sustained-release implants. Transl Vis Sci Technol.

[21] Seal JR, Robinson MR, Burke J, Bejanian M, Coote M, Attar M (2019). Intracameral sustained-release bimatoprost implant delivers bimatoprost to target tissues with reduced drug exposure to off-target tissues. J Ocul Pharmacol Ther.

[22] Medeiros FA, Walters TR, Kolko M (2020). Phase 3, randomized, 20-month study of bimatoprost implant in open-angle glaucoma and ocular hypertension (ARTEMIS 1). Ophthalmology.

[23] Bacharach J, Tatham A, Ferguson G (2021). Phase 3, randomized, 20-month study of the efficacy and safety of bimatoprost implant in patients with open-angle glaucoma and ocular hypertension (ARTEMIS 2). Drugs.

[24] Craven ER, Walters T, Christie WC (2020). 24-month phase I/II clinical trial of bimatoprost sustained-release implant (Bimatoprost SR) in glaucoma patients. Drugs.

[25] Kolko M, Tatham AJ, Lim KS (2025). Phase 3, randomized, comparison study of intracameral bimatoprost implant 10 µg and selective laser trabeculoplasty. Am J Ophthalmol.

[26] Teymoorian S, Craven ER, Nguyen L, Werts E (2024). Real-world study of the effectiveness and safety of intracameral bimatoprost implant in a clinical setting in the united states. Clin Ophthalmol.

[27] Ali AA, Avilés Elescano D, Grover DS (2024). Bimatoprost SR for glaucoma therapy implanted at the slit-lamp in a real-world setting. Clin Ophthalmol.

[28] Franca JR, Foureaux G, Fuscaldi LL (2014). Bimatoprost-loaded ocular inserts as sustained release drug delivery systems for glaucoma treatment: in vitro and in vivo evaluation. PLoS One.

[29] Yadav M, Guzman-Aranguez A, Perez de Lara MJ, Singh M, Singh J, Kaur IP (2019). Bimatoprost loaded nanovesicular long-acting sub-conjunctival in-situ gelling implant: in vitro and in vivo evaluation. Mater Sci Eng C Mater Biol Appl.

